# Crystal structure of the Anderson-type hetero­polyoxometalate; K_2_[H_7_Cr^III^Mo_6_O_24_]·8H_2_O: a redetermination revealing the position of the extra H atom in the polyanion

**DOI:** 10.1107/S2056989015000390

**Published:** 2015-01-17

**Authors:** Hea-Chung Joo, Ki-Min Park, Uk Lee

**Affiliations:** a Department of Chemistry, Pukyong National University, 599-1 Daeyeon 3-dong, Nam-gu, Busan 608-737, Republic of Korea; b The Research Institute of Natural Science, Gyeongsan National University, Jinju 660-701, Republic of Korea

**Keywords:** crystal structure, redetermination, B-series Anderson-type polyanion, symmetric hydrogen bond

## Abstract

The title compound contains a symmetric hydrogen bond in which the H atom does not lie on a crystallographic centre of symmetry. The structure of K_2_[H_7_Cr^III^Mo_6_O_24_]·8H_2_O, namely dipotassium hepta­hydrogen hexa­molybdochromate(III) octa­hydrate, previously reported by Lee [*Acta Cryst*. (2007), E**63**, i5–i7], has been redetermined in order to locate the position of the seventh H atom in the anion. Six of the H atoms are bonded to the six μ_3_-O atoms and form hydrogen bonds of medium strength either to water mol­ecules or to the terminal O atoms of other polyanions. The seventh H atom forms a very short hydrogen bond between two μ_2_-O atoms on adjacent polyanions. This short bond, together with two normal hydrogen bonds, link the two crystallographically distinct centrosymmetric polyanions into chains along [011], while the length of this bond [2.461 (3) Å] suggests that the H atom lies at its centre, but unusually for such a bond, this point is not a crystallographic centre of symmetry.

## Abstract 2

K_2_[H_7_Cr^III^Mo_6_O_24_]·8H_2_O contains a symmetric hydrogen bond in which the H atom does not lie on a crystallographic centre of symmetry. The structure has been redetermined in order to locate the position of the seventh H atom.

## Chemical context   

This redetermined structure of a typical Anderson-type heteropolyoxometalate (Anderson, 1937 ▸), K_2_[H_7_Cr^III^Mo_6_O_24_]·8H_2_O reveals the position of the extra or seventh H atom in the [HCr^III^(OH)_6_Mo_6_O_18_]^2−^ polyanion. This has not only an extra H atom but this atom also forms a very short hydrogen bond [2.461 (3) Å]; however, the H atom that contributes to the short hydrogen bond does not lie on a crystallographic centre of symmetry.

An example of a relatively short hydrogen bond in which the H atom does lie on a crystallographic centre of symmetry in an Anderson-type polyanion was reported in the polyoxometalate, *viz*. K_7_[H_4.5_α-PtMo_6_O_24_]_2_·11H_2_O (Lee *et al.*, 2010 ▸). In this compound, the two polyanions form a dimer, *viz*. [(H_4.5_PtMo_6_O_24_)_2_]^7−^ *via* seven hydrogen bonds, *viz*. four μ_3_-O—H⋯μ_1_–O (terminal Mo=O atom), two μ_2_-O—H⋯μ_2_-O and one central/symmetric μ_3_-O⋯H⋯μ_3_-O. The H atom of the central hydrogen bond lies on a crystallographic centre of symmetry (space group *P* 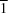: ½, 0, ½) with a μ_3_-O⋯H⋯μ_3_-O (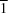) distance of 2.553 (3) Å. In this way, the hydrogen bond is symmetric, O⋯H⋯O, and the donor and acceptor cannot be distinguished. The dimerization of the polyanion by these hydrogen bonds is possible because the μ_3_-O atoms in the polyanion are only partially protonated. The location of the H atom in the central μ_3_-O⋯H⋯μ_3_-O unit was determined from a centrosymmetric electron density map around the H-atom position. This centrosymmetric inter­pretation of the hydrogen bond is strongly supported by the bond-valence sums (BVS; Brown & Altermatt, 1985 ▸; Brese & O’Keeffe, 1991 ▸). The sum around the strongly bonded μ_3_-O atom is 1.92 valence units (v.u.) in the [(H_4.5_PtMo_6_O_24_)_2_]^7−^ polyanion. The reasonable BVS values of very short or very long O—H bond distances can be obtained from the graphical correlation (Brown, 2001 ▸).

**Figure 1:**
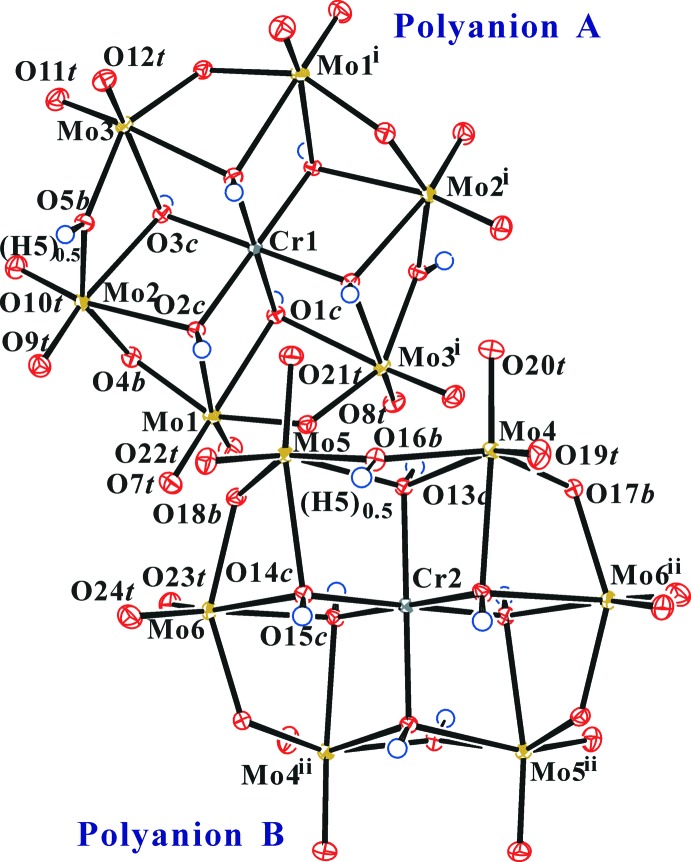
The polyanion structure in the title compound. Displacement ellipsoids are drawn at the 50% probability level for non-H atoms. H atoms are shown as small spheres of arbitrary radius. [Symmetry codes: (i) −*x* + 1, −*y*, −*z* + 2; (ii) −*x* + 1, −*y* + 1, −*z* + 1.]

However, the title compound belongs to the B-series Anderson-type polyanions (Tsigdinos, 1978 ▸) *viz*. [*X^n^* ^+^(OH)_6_MoO_18_]^(12−*n*)−^ (*X* = heteroatom), in which such dimerization is impossible because all six μ_3_-O atoms are fully protonated. The polyanion structure in the title compound is shown in Fig. 1 ▸.

## Structural commentary   

**Figure 2:**
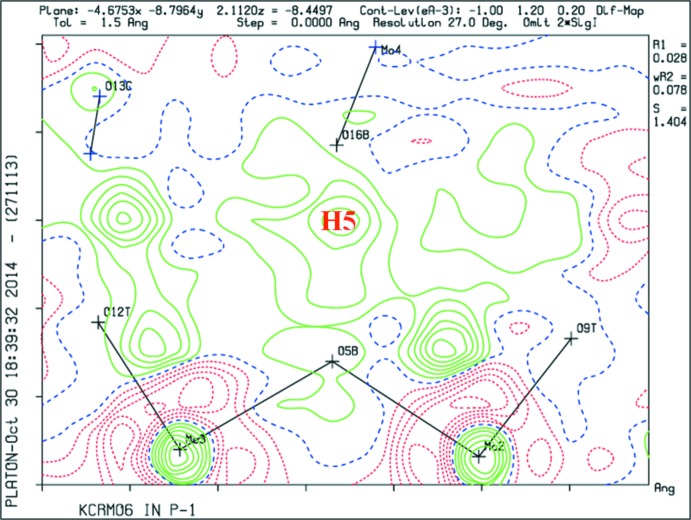
Difference Fourier map around atom H5 where atom H5 is absent.

This study was carried out to clearly identify the position of the seventh or extra H atom in the [HCr^III^(OH)_6_Mo_6_O_18_]^2−^ polyanion. After considering the electron density maps and BVS values of the protonated O*B* (O-bridged μ_2_-O atom) atoms in the previously reported structure (Lee, 2007 ▸), we came to the conclusion that the positional disorder model of the H atom was wrong. The electron density (Fig. 2 ▸) is not symmetric in the title compound, but we expect the H atoms to lie in the middle of the bond because of the short O⋯O distance of 2.461 (3) Å, which corresponds to a pseudosymmetric hydrogen bond. The description of the rest of the structure and the composition of the atoms in the polyanion are the same as in the previous report of the compound, *viz*. K_2_[H_7_Cr^III^Mo_6_O_24_]·8H_2_O (Lee, 2007 ▸). The O atoms of the polyoxometalate are designated as O*T* (terminal Mo=O atom), O*B*, and O*C* (centre of two Mo and one Cr atom, μ_3_-O atom), respectively.

In the present case, the O5*B*⋯H5 and O16*B* ^i^⋯H5 distances are both 1.23 Å (Table 1 ▸). However, since the H atom does not lie on a crystallographic centre of symmetry, the present structure is considered to be particularly significant. As a result, the H5 atom is co-shared as O5*B*⋯H5⋯O16*B* ^i^, and the average equation of the polyanion is [Cr^III^(μ_3_-OH)_6_{μ_2_-O(0.5H)}_2_Mo_6_O_17_]^2−^.

The calculated BVS for the O5*B* and O16*B* atoms are 1.59 and 1.57 v.u., respectively, if the valence of the O—H bond is not included. Since the BVS value around the μ_2_-O atom should be 2.0 v.u., the missing valences of O5*B* and O16*B* are 0.41 and 0.43 v.u., respectively, corresponding to the valence of the O—H bonds. The obtained graphical correlation valence of H5 from its distance, 1.232 (7) Å, is 0.41 v.u., which is sufficient to satisfy the sums around the O5*B* and O16*B* atoms. As a result, the valence sums around O5*B* and O16*B* are 2.00 and 1.98 v.u., respectively. The BVS around the unprotonated μ_2_-O atoms, *viz*. O4*B*, O6*B*, O17*B* and O18*B* are 1.98, 1.96, 2.13 and 2.02 v.u., respectively.

The positional disordered model in the previous report (Lee, 2007 ▸) showed unreasonable BVS values. The calculated BVS for the O5*B* and O16*B* atoms are 1.62 and 1.57 v.u., respectively, if the valence of the O—H bond is not included. The obtained graphical correlation valences of H5 and H16 from its distances [O5*B*—H5 = 0.71 (8), H5⋯O16*B* = 1.79 (8) Å and O16*B*—H16 = 0.83 (8), H16⋯O5*B* = 1.65 (8) Å] are 0.24 and 0.26 v.u. Therefore, the total BVS values of O5*B* and O16*B* are 1.86 and 1.83 v.u., respectively.

As a result, we consider that the present model of the title compound is more reasonable, and the one extra H atom is located at the mid-point between the O5*B* and O16*B* atoms, and shared equally by two discrete polyanions. All H atoms and hydrogen bonds are well-defined in the title compound (Table 1 ▸).

## Supra­molecular features   

**Figure 3:**
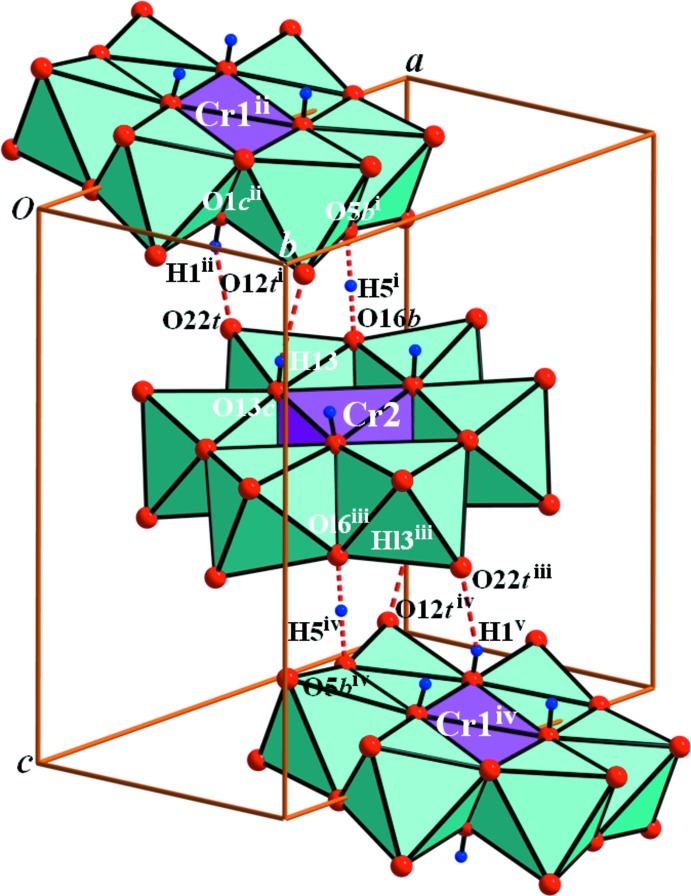
Polyhedral view of the heteropolyanion in (I), with O⋯O contacts of the inter-polyanion hydrogen bonds shown as dashed lines. [Symmetry codes; (i) −*x* + 1, −*y*, −*z* + 1; (ii) *x*, *y*, *z* − 1; (iii) −*x* + 1, −*y* + 1, −*z* + 1; (iv) *x*, *y* + 1, *z*; (v) −*x* + 1, −*y* + 1, −*z* + 2.]

Two discrete polyanions *A* and *B* are linked into chains along [011] by two normal, and one strong and pseudosymmetric hydrogen bonds (Table 1 ▸ and Fig. 3 ▸). The K^+^ ions are variously coordinated by O atoms as [K1(O*T*)_4_(O*W*)_4_]^+^ and [K2(O*T*)_4_(O*B*)(O*W*)_3_]^+^ in the distance range 2.722 (3)–3.075 (3) Å. Furthermore, the polyanions are three dimensionally linked *via* K⋯O*T* inter­actions. All water mol­ecules form hydrogen bonds with polyanions except for the O30*W* and O31*W* water mol­ecules.

## Synthesis and crystallization   

The crude potassium salt of title compound was obtained from the reaction of an Na_3_[H_6_CrMo_6_O_24_]·8H_2_O (Perloff, 1970 ▸) solution and excess KCl solution. The title compound was obtained by recrystallization of crude K_3_[H_6_CrMo_6_O_24_]·8H_2_O at pH 1.80.

## Refinement   

Crystal data, data collection and structure refinement details are summarized in Table 2 ▸. All the H atoms in the polyanions and all water H atoms were positioned using difference Fourier maps. All H atoms in the polyanion were refined freely, but the H5 atoms were refined with a distance restraint of O5*B*—H5 and O16*B* ^iii^—H5 (Table 1 ▸) using the SADI command in *SHELXL97* (Sheldrick, 2008 ▸); σ = 0.01, the distances between the first and second named bonds were restrained to be equal with an effective standard deviation sigma in order to locate the H5 atom on the pseudocentre between the O5*B* and O16*B* atoms. The H atoms of all water mol­ecules (O*W*) were refined with a distance restraint of O—H = 0.95 (3) Å using the DFIX command, and were included in the refinement with *U*_iso_(H) = 1.5*U*_eq_(O). The highest peak in the difference map is 1.62 Å from O9*T*.

## Supplementary Material

Crystal structure: contains datablock(s) I. DOI: 10.1107/S2056989015000390/br2242sup1.cif

Structure factors: contains datablock(s) I. DOI: 10.1107/S2056989015000390/br2242Isup2.hkl

CCDC reference: 1042715

Additional supporting information:  crystallographic information; 3D view; checkCIF report

## Figures and Tables

**Table 1:** Hydrogen-bond geometry (, )

*D*H*A*	*D*H	H*A*	*D* *A*	*D*H*A*
O1*C*H1O22*T* ^i^	0.70(6)	2.11(6)	2.789(4)	164(6)
O2*C*H2O27*W*	0.76(6)	2.01(6)	2.753(4)	166(6)
O3*C*H3O31*W* ^ii^	0.82(6)	1.79(6)	2.604(4)	176(6)
O5*B*H5O16*B* ^iii^	1.23(1)	1.23(1)	2.461(3)	175(5)
O13*C*H13O12*T* ^iii^	0.70(5)	2.05(5)	2.734(4)	167(5)
O14*C*H14O32*W*	0.81(5)	2.01(5)	2.776(4)	158(5)
O15*C*H15O28*W* ^iv^	0.76(6)	1.87(6)	2.619(4)	170(6)
O25*W*H25*A*O21*T*	0.92(3)	1.96(3)	2.838(4)	157(5)
O25*W*H25*B*O20*T*	0.93(3)	1.92(3)	2.819(4)	162(5)
O26*W*H26*A*O4*B* ^v^	0.91(3)	1.89(3)	2.748(4)	158(5)
O26*W*H26*B*O24*T* ^iii^	0.91(3)	1.91(3)	2.798(4)	164(5)
O27*W*H27*A*O18*B*	0.90(3)	1.95(4)	2.775(4)	151(5)
O27*W*H27*B*O32*W*	0.91(3)	1.92(3)	2.825(5)	171(5)
O28*W*H28*A*O12*T* ^vi^	0.91(3)	2.00(4)	2.785(4)	144(5)
O28*W*H28*B*O31*W* ^iv^	0.90(3)	1.82(3)	2.706(4)	170(5)
O29*W*H29*A*O11*T* ^v^	0.95(3)	2.11(4)	2.949(5)	146(6)
O29*W*H29*B*O8*T* ^vii^	0.96(3)	2.18(3)	3.126(5)	169(6)
O31*W*H31*A*O26*W* ^iv^	0.90(3)	1.77(3)	2.665(4)	170(5)
O31*W*H31*B*O27*W* ^iv^	0.92(3)	2.18(4)	2.952(4)	141(4)
O32*W*H32*A*O30*W*	0.89(3)	2.06(3)	2.947(6)	171(5)
O32*W*H32*B*O6*B* ^v^	0.89(3)	2.39(5)	3.000(4)	126(5)

Symmetry codes: (i) 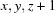; (ii) 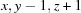; (iii) 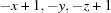; (iv) 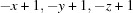; (v) 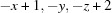; (vi) 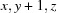; (vii) 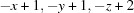.

**Table 2:** Experimental details

Crystal data	
Chemical formula	K_2_[H_7_CrMo_6_O_24_]8H_2_O
*M*_r_	1241.02
Crystal system, space group	Triclinic, *P* 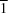
Temperature (K)	446
*a*, *b*, *c* ()	10.4588(2), 10.8553(2), 12.6287(3)
, , ()	99.296(1), 94.469(1), 99.283(1)
*V* (^3^)	1388.44(5)
*Z*	2
Radiation type	Mo *K*
(mm^1^)	3.42
Crystal size (mm)	0.18 0.11 0.09

Data collection	
Diffractometer	Bruker *SMART* APEXII CCD
Absorption correction	Multi-scan (*SADABS*; Sheldrick, 2008 ▸)
*T*_min_, *T*_max_	0.645, 0.746
No. of measured, independent and observed [*I* > 2(*I*)] reflections	23489, 6028, 5893
*R*_int_	0.025
(sin /)_max_ (^1^)	0.639

Refinement	
*R*[*F* ^2^ > 2(*F* ^2^)], *wR*(*F* ^2^), *S*	0.024, 0.064, 1.11
No. of reflections	6028
No. of parameters	450
No. of restraints	17
H-atom treatment	H atoms treated by a mixture of independent and constrained refinement
_max_, _min_ (e ^3^)	1.58, 0.67

Computer programs: *APEX2* and *SAINT* (Bruker, 2009 ▸), *SHELXS97* and *SHELXL97* (Sheldrick, 2008 ▸), *ORTEP-3 for Windows* (Farrugia, 2012 ▸), *PLATON* (Spek, 2009 ▸) and *DIAMOND* (Brandenburg, 1998 ▸).

## supplementary crystallographic information

### Crystal data

**Table:**

K_2_[H_7_CrMo_6_O_24_]·8H_2_O	*Z* = 2
*M**_r_* = 1241.02	*F*(000) = 1186
Triclinic, *P*1	*D*_x_ = 2.968 Mg m^−^^3^
Hall symbol: -P 1	Mo *K*α radiation, λ = 0.71073 Å
*a* = 10.4588 (2) Å	Cell parameters from 9896 reflections
*b* = 10.8553 (2) Å	θ = 2.5–33.5°
*c* = 12.6287 (3) Å	µ = 3.42 mm^−^^1^
α = 99.296 (1)°	*T* = 446 K
β = 94.469 (1)°	Block, purple
γ = 99.283 (1)°	0.18 × 0.11 × 0.09 mm
*V* = 1388.44 (5) Å^3^	

### Data collection

**Table:**

Bruker SMART APEXII CCD diffractometer	6028 independent reflections
Radiation source: Rotating Anode	5893 reflections with *I* > 2σ(*I*)
Graphite multilayer monochromator	*R*_int_ = 0.025
Detector resolution: 10.0 pixels mm^-1^	θ_max_ = 27.0°, θ_min_ = 1.6°
φ and ω scans	*h* = −13→13
Absorption correction: multi-scan (*SADABS*; Sheldrick, 2008)	*k* = −13→13
*T*_min_ = 0.645, *T*_max_ = 0.746	*l* = −16→16
23489 measured reflections	

### Refinement

**Table:**

Refinement on *F*^2^	Secondary atom site location: difference Fourier map
Least-squares matrix: full	Hydrogen site location: difference Fourier map
*R*[*F*^2^ > 2σ(*F*^2^)] = 0.024	H atoms treated by a mixture of independent and constrained refinement
*wR*(*F*^2^) = 0.064	*w* = 1/[σ^2^(*F*_o_^2^) + (0.0255*P*)^2^ + 6.3059*P*] where *P* = (*F*_o_^2^ + 2*F*_c_^2^)/3
*S* = 1.11	(Δ/σ)_max_ = 0.001
6028 reflections	Δρ_max_ = 1.58 e Å^−^^3^
450 parameters	Δρ_min_ = −0.67 e Å^−^^3^
17 restraints	Extinction correction: *SHELXL97* (Sheldrick, 2008), Fc^*^=kFc[1+0.001xFc^2^λ^3^/sin(2θ)]^-1/4^
Primary atom site location: structure-invariant direct methods	Extinction coefficient: 0.00391 (16)

### Special details

**Table:**

Geometry. All esds (except the esd in the dihedral angle between two l.s. planes) are estimated using the full covariance matrix. The cell esds are taken into account individually in the estimation of esds in distances, angles and torsion angles; correlations between esds in cell parameters are only used when they are defined by crystal symmetry. An approximate (isotropic) treatment of cell esds is used for estimating esds involving l.s. planes.
Refinement. Refinement of F^2^ against ALL reflections. The weighted R-factor wR and goodness of fit S are based on F^2^, conventional R-factors R are based on F, with F set to zero for negative F^2^. The threshold expression of F^2^ > 2sigma(F^2^) is used only for calculating R-factors(gt) etc. and is not relevant to the choice of reflections for refinement. R-factors based on F^2^ are statistically about twice as large as those based on F, and R- factors based on ALL data will be even larger.

### Fractional atomic coordinates and isotropic or equivalent isotropic displacement parameters (Å^2^)

**Table:**

	*x*	*y*	*z*	*U*_iso_*/*U*_eq_	
Cr1	0.5000	0.0000	1.0000	0.00813 (15)	
Cr2	0.5000	0.5000	0.5000	0.00762 (15)	
Mo1	0.23267 (3)	0.13627 (3)	0.99546 (2)	0.01127 (8)	
Mo2	0.20792 (3)	−0.17249 (3)	0.90598 (2)	0.01086 (8)	
Mo3	0.47636 (3)	−0.31129 (3)	0.92212 (2)	0.01051 (8)	
Mo4	0.79232 (3)	0.42389 (3)	0.46268 (2)	0.00994 (8)	
Mo5	0.52144 (3)	0.19391 (3)	0.43518 (2)	0.00938 (8)	
Mo6	0.23147 (3)	0.27465 (3)	0.46825 (2)	0.00952 (8)	
K1	0.06894 (8)	0.02275 (9)	0.66878 (7)	0.02014 (17)	
K2	0.92835 (8)	0.58495 (9)	0.77823 (7)	0.02212 (18)	
O1C	0.4157 (2)	0.1138 (2)	1.0986 (2)	0.0113 (5)	
H1	0.414 (6)	0.100 (5)	1.151 (5)	0.033 (16)*	
O2C	0.3526 (2)	0.0070 (2)	0.8952 (2)	0.0099 (5)	
H2	0.362 (5)	0.029 (5)	0.842 (4)	0.028 (14)*	
O3C	0.3916 (2)	−0.1598 (2)	1.0194 (2)	0.0109 (5)	
H3	0.386 (5)	−0.166 (5)	1.083 (5)	0.034 (15)*	
O4B	0.1720 (2)	−0.0378 (2)	1.0107 (2)	0.0137 (5)	
O5B	0.3370 (2)	−0.2592 (2)	0.83029 (19)	0.0121 (5)	
H5	0.334 (9)	−0.271 (9)	0.7314 (10)	0.12 (3)*	
O6B	0.6225 (2)	−0.2660 (2)	1.0259 (2)	0.0131 (5)	
O7T	0.1329 (3)	0.1463 (3)	0.8855 (2)	0.0196 (6)	
O8T	0.1748 (3)	0.2124 (3)	1.1047 (2)	0.0201 (6)	
O9T	0.1160 (3)	−0.1627 (3)	0.7911 (2)	0.0171 (5)	
O10T	0.1270 (3)	−0.2975 (3)	0.9535 (2)	0.0192 (6)	
O11T	0.3862 (3)	−0.4281 (3)	0.9735 (2)	0.0184 (5)	
O12T	0.5387 (3)	−0.3860 (2)	0.8119 (2)	0.0158 (5)	
O13C	0.4147 (2)	0.3516 (2)	0.3922 (2)	0.0099 (5)	
H13	0.415 (5)	0.359 (5)	0.338 (4)	0.015 (12)*	
O14C	0.3526 (2)	0.4487 (2)	0.5815 (2)	0.0100 (5)	
H14	0.357 (5)	0.437 (5)	0.643 (4)	0.019 (12)*	
O15C	0.3920 (2)	0.6197 (2)	0.4556 (2)	0.0096 (5)	
H15	0.376 (5)	0.616 (5)	0.395 (5)	0.034 (15)*	
O16B	0.6638 (2)	0.2882 (2)	0.36714 (19)	0.0110 (5)	
O17B	0.8278 (2)	0.5773 (2)	0.56733 (19)	0.0113 (5)	
O18B	0.3755 (2)	0.1934 (2)	0.5151 (2)	0.0116 (5)	
O19T	0.8822 (3)	0.4581 (3)	0.3615 (2)	0.0179 (5)	
O20T	0.8741 (3)	0.3335 (3)	0.5323 (2)	0.0187 (6)	
O21T	0.6089 (2)	0.1094 (3)	0.5064 (2)	0.0165 (5)	
O22T	0.4583 (3)	0.0957 (2)	0.3159 (2)	0.0157 (5)	
O23T	0.1361 (2)	0.2368 (3)	0.5654 (2)	0.0155 (5)	
O24T	0.1681 (3)	0.1718 (3)	0.3527 (2)	0.0176 (5)	
O25W	0.8716 (3)	0.0732 (3)	0.5305 (2)	0.0206 (6)	
H25A	0.783 (3)	0.069 (5)	0.535 (4)	0.031*	
H25B	0.891 (5)	0.159 (3)	0.530 (4)	0.031*	
O26W	0.8483 (3)	0.0653 (3)	0.7784 (2)	0.0235 (6)	
H26A	0.863 (5)	0.051 (5)	0.847 (3)	0.035*	
H26B	0.827 (5)	−0.012 (3)	0.735 (4)	0.035*	
O27W	0.3568 (3)	0.1168 (3)	0.7134 (2)	0.0261 (7)	
H27A	0.367 (6)	0.113 (5)	0.643 (2)	0.039*	
H27B	0.337 (6)	0.194 (3)	0.739 (4)	0.039*	
O28W	0.6802 (3)	0.4221 (3)	0.7521 (2)	0.0208 (6)	
H28A	0.629 (4)	0.464 (5)	0.794 (4)	0.031*	
H28B	0.669 (5)	0.345 (3)	0.770 (4)	0.031*	
O29W	0.8747 (4)	0.5437 (4)	0.9821 (3)	0.0390 (8)	
H29A	0.810 (5)	0.479 (5)	0.998 (5)	0.059*	
H29B	0.851 (6)	0.611 (5)	0.948 (5)	0.059*	
O30W	0.0221 (5)	0.3490 (4)	0.7849 (5)	0.0640 (14)	
H30A	−0.019 (9)	0.333 (9)	0.716 (4)	0.096*	
H30B	−0.022 (8)	0.268 (4)	0.786 (7)	0.096*	
O31W	0.3602 (3)	0.8223 (3)	0.2192 (2)	0.0206 (6)	
H31A	0.295 (4)	0.868 (5)	0.226 (4)	0.031*	
H31B	0.432 (4)	0.870 (4)	0.261 (4)	0.031*	
O32W	0.3044 (3)	0.3645 (3)	0.7732 (2)	0.0245 (6)	
H32A	0.219 (3)	0.366 (5)	0.772 (4)	0.037*	
H32B	0.346 (5)	0.392 (5)	0.839 (3)	0.037*	

### Atomic displacement parameters (Å^2^)

**Table:**

	*U*^11^	*U*^22^	*U*^33^	*U*^12^	*U*^13^	*U*^23^
Cr1	0.0082 (3)	0.0076 (4)	0.0084 (3)	0.0010 (3)	0.0006 (3)	0.0013 (3)
Cr2	0.0070 (3)	0.0074 (4)	0.0086 (3)	0.0013 (3)	0.0009 (3)	0.0015 (3)
Mo1	0.01095 (14)	0.01186 (15)	0.01156 (14)	0.00390 (11)	0.00131 (11)	0.00177 (11)
Mo2	0.00957 (14)	0.01067 (15)	0.01165 (14)	0.00013 (11)	0.00034 (10)	0.00198 (11)
Mo3	0.01252 (14)	0.00810 (15)	0.01083 (14)	0.00194 (11)	0.00182 (11)	0.00109 (11)
Mo4	0.00749 (13)	0.00962 (15)	0.01315 (14)	0.00208 (10)	0.00177 (10)	0.00245 (11)
Mo5	0.00942 (14)	0.00756 (14)	0.01113 (14)	0.00169 (10)	0.00103 (10)	0.00138 (10)
Mo6	0.00820 (14)	0.00838 (15)	0.01158 (14)	0.00012 (10)	0.00090 (10)	0.00201 (11)
K1	0.0189 (4)	0.0248 (4)	0.0182 (4)	0.0039 (3)	0.0019 (3)	0.0081 (3)
K2	0.0173 (4)	0.0303 (5)	0.0177 (4)	0.0012 (3)	0.0017 (3)	0.0040 (3)
O1C	0.0135 (12)	0.0115 (12)	0.0089 (12)	0.0027 (9)	0.0016 (9)	0.0011 (10)
O2C	0.0108 (11)	0.0116 (12)	0.0081 (11)	0.0022 (9)	0.0019 (9)	0.0036 (9)
O3C	0.0135 (12)	0.0103 (12)	0.0093 (11)	0.0018 (9)	0.0017 (9)	0.0024 (9)
O4B	0.0130 (12)	0.0148 (13)	0.0135 (12)	0.0022 (10)	0.0041 (9)	0.0022 (10)
O5B	0.0127 (12)	0.0128 (12)	0.0106 (11)	0.0036 (9)	0.0005 (9)	0.0004 (9)
O6B	0.0144 (12)	0.0117 (12)	0.0129 (12)	0.0024 (10)	−0.0009 (9)	0.0026 (9)
O7T	0.0159 (13)	0.0214 (15)	0.0212 (14)	0.0031 (11)	−0.0022 (10)	0.0058 (11)
O8T	0.0206 (14)	0.0200 (14)	0.0203 (13)	0.0046 (11)	0.0066 (11)	0.0019 (11)
O9T	0.0168 (13)	0.0182 (14)	0.0157 (13)	0.0024 (11)	−0.0004 (10)	0.0028 (10)
O10T	0.0184 (13)	0.0168 (14)	0.0230 (14)	0.0013 (11)	0.0040 (11)	0.0062 (11)
O11T	0.0199 (13)	0.0145 (13)	0.0219 (13)	0.0023 (11)	0.0037 (11)	0.0063 (11)
O12T	0.0198 (13)	0.0140 (13)	0.0136 (12)	0.0047 (10)	0.0029 (10)	0.0002 (10)
O13C	0.0118 (12)	0.0104 (12)	0.0073 (12)	0.0014 (9)	0.0006 (9)	0.0019 (9)
O14C	0.0086 (11)	0.0130 (12)	0.0088 (11)	0.0014 (9)	0.0007 (9)	0.0039 (9)
O15C	0.0110 (11)	0.0096 (12)	0.0082 (11)	0.0019 (9)	−0.0002 (9)	0.0021 (9)
O16B	0.0122 (11)	0.0120 (12)	0.0083 (11)	0.0014 (9)	0.0033 (9)	0.0003 (9)
O17B	0.0103 (11)	0.0106 (12)	0.0124 (11)	0.0013 (9)	−0.0005 (9)	0.0022 (9)
O18B	0.0112 (11)	0.0106 (12)	0.0140 (12)	0.0022 (9)	0.0029 (9)	0.0045 (9)
O19T	0.0144 (12)	0.0169 (14)	0.0225 (14)	0.0003 (10)	0.0089 (10)	0.0031 (11)
O20T	0.0147 (13)	0.0159 (14)	0.0263 (14)	0.0055 (10)	−0.0019 (11)	0.0055 (11)
O21T	0.0127 (12)	0.0150 (13)	0.0240 (14)	0.0046 (10)	0.0028 (10)	0.0075 (11)
O22T	0.0162 (12)	0.0136 (13)	0.0156 (12)	0.0005 (10)	0.0014 (10)	0.0001 (10)
O23T	0.0139 (12)	0.0150 (13)	0.0187 (13)	0.0008 (10)	0.0049 (10)	0.0065 (10)
O24T	0.0165 (13)	0.0187 (14)	0.0168 (13)	0.0050 (11)	−0.0018 (10)	0.0005 (10)
O25W	0.0178 (13)	0.0182 (14)	0.0278 (15)	0.0071 (11)	0.0016 (11)	0.0062 (12)
O26W	0.0302 (16)	0.0238 (16)	0.0165 (13)	0.0047 (13)	0.0058 (12)	0.0023 (12)
O27W	0.0351 (17)	0.0310 (17)	0.0174 (14)	0.0118 (14)	0.0055 (12)	0.0121 (13)
O28W	0.0271 (15)	0.0180 (14)	0.0195 (14)	0.0085 (12)	0.0042 (11)	0.0050 (11)
O29W	0.040 (2)	0.040 (2)	0.043 (2)	0.0083 (16)	0.0114 (16)	0.0201 (17)
O30W	0.046 (3)	0.038 (2)	0.117 (4)	0.015 (2)	0.013 (3)	0.030 (3)
O31W	0.0258 (15)	0.0192 (15)	0.0157 (13)	0.0010 (12)	0.0010 (11)	0.0034 (11)
O32W	0.0257 (15)	0.0305 (17)	0.0152 (13)	−0.0007 (13)	0.0019 (12)	0.0036 (12)

### Geometric parameters (Å, º)

**Table:**

Cr1—O1C^i^	1.970 (2)	Mo6—O18B	1.964 (2)
Cr1—O1C	1.970 (3)	Mo6—O14C	2.301 (2)
Cr1—O2C	1.971 (2)	Mo6—O13C	2.307 (3)
Cr1—O2C^i^	1.971 (2)	K1—O25W^iii^	2.722 (3)
Cr1—O3C^i^	1.975 (2)	K1—O25W^iv^	2.778 (3)
Cr1—O3C	1.975 (2)	K1—O9T	2.809 (3)
Cr2—O13C^ii^	1.970 (2)	K1—O7T	2.825 (3)
Cr2—O13C	1.970 (2)	K1—O26W^iv^	2.844 (3)
Cr2—O15C	1.972 (2)	K1—O23T	2.862 (3)
Cr2—O15C^ii^	1.972 (2)	K1—O24T^v^	2.950 (3)
Cr2—O14C^ii^	1.976 (2)	K1—O27W	2.997 (3)
Cr2—O14C	1.976 (2)	K2—O29W	2.767 (4)
Mo1—O8T	1.696 (3)	K2—O17B	2.769 (3)
Mo1—O7T	1.699 (3)	K2—O19T^vi^	2.799 (3)
Mo1—O4B	1.939 (3)	K2—O8T^vii^	2.855 (3)
Mo1—O6B^i^	1.962 (3)	K2—O28W	2.858 (3)
Mo1—O1C	2.299 (3)	K2—O10T^viii^	2.891 (3)
Mo1—O2C	2.321 (2)	K2—O30W^ix^	2.897 (4)
Mo2—O10T	1.701 (3)	K2—O9T^viii^	3.074 (3)
Mo2—O9T	1.705 (3)	O1C—H1	0.70 (6)
Mo2—O4B	1.913 (3)	O2C—H2	0.76 (6)
Mo2—O5B	1.986 (2)	O3C—H3	0.82 (6)
Mo2—O3C	2.276 (2)	O5B—O16B^iii^	2.461 (3)
Mo2—O2C	2.296 (3)	O5B—H5	1.232 (7)
Mo3—O11T	1.701 (3)	O13C—H13	0.70 (5)
Mo3—O12T	1.722 (3)	O14C—H14	0.81 (5)
Mo3—O6B	1.879 (2)	O15C—H15	0.76 (6)
Mo3—O5B	2.001 (2)	O16B—H5^iii^	1.231 (7)
Mo3—O3C	2.233 (2)	O25W—H25A	0.92 (3)
Mo3—O1C^i^	2.321 (3)	O25W—H25B	0.93 (3)
Mo4—O19T	1.698 (3)	O26W—H26A	0.91 (3)
Mo4—O20T	1.702 (3)	O26W—H26B	0.91 (3)
Mo4—O17B	1.916 (2)	O27W—H27A	0.90 (3)
Mo4—O16B	1.992 (2)	O27W—H27B	0.91 (3)
Mo4—O15C^ii^	2.275 (2)	O28W—H28A	0.91 (3)
Mo4—O14C^ii^	2.306 (2)	O28W—H28B	0.90 (3)
Mo5—O21T	1.703 (3)	O29W—H29A	0.95 (3)
Mo5—O22T	1.715 (3)	O29W—H29B	0.96 (3)
Mo5—O18B	1.894 (2)	O30W—H30A	0.92 (3)
Mo5—O16B	1.998 (2)	O30W—H30B	0.93 (3)
Mo5—O15C^ii^	2.264 (2)	O31W—H31A	0.90 (3)
Mo5—O13C	2.301 (2)	O31W—H31B	0.92 (3)
Mo6—O23T	1.700 (3)	O32W—H32A	0.89 (3)
Mo6—O24T	1.703 (3)	O32W—H32B	0.89 (3)
Mo6—O17B^ii^	1.919 (2)		
			
O1C^i^—Cr1—O1C	180.00 (13)	O23T—Mo6—O24T	106.46 (13)
O1C^i^—Cr1—O2C	96.36 (11)	O23T—Mo6—O17B^ii^	102.39 (12)
O1C—Cr1—O2C	83.64 (10)	O24T—Mo6—O17B^ii^	98.17 (12)
O1C^i^—Cr1—O2C^i^	83.64 (10)	O23T—Mo6—O18B	95.85 (11)
O1C—Cr1—O2C^i^	96.36 (11)	O24T—Mo6—O18B	100.31 (12)
O2C—Cr1—O2C^i^	180.000 (1)	O17B^ii^—Mo6—O18B	149.10 (10)
O1C^i^—Cr1—O3C^i^	96.40 (11)	O23T—Mo6—O14C	92.72 (11)
O1C—Cr1—O3C^i^	83.60 (11)	O24T—Mo6—O14C	160.17 (11)
O2C—Cr1—O3C^i^	96.11 (10)	O17B^ii^—Mo6—O14C	72.22 (9)
O2C^i^—Cr1—O3C^i^	83.89 (10)	O18B—Mo6—O14C	82.29 (10)
O1C^i^—Cr1—O3C	83.60 (11)	O23T—Mo6—O13C	158.88 (11)
O1C—Cr1—O3C	96.40 (11)	O24T—Mo6—O13C	92.55 (11)
O2C—Cr1—O3C	83.89 (10)	O17B^ii^—Mo6—O13C	83.47 (10)
O2C^i^—Cr1—O3C	96.11 (10)	O18B—Mo6—O13C	71.21 (9)
O3C^i^—Cr1—O3C	180.0	O14C—Mo6—O13C	69.50 (9)
O13C^ii^—Cr2—O13C	180.00 (11)	O25W^iii^—K1—O25W^iv^	76.62 (9)
O13C^ii^—Cr2—O15C	83.38 (10)	O25W^iii^—K1—O9T	103.15 (9)
O13C—Cr2—O15C	96.62 (10)	O25W^iv^—K1—O9T	139.32 (9)
O13C^ii^—Cr2—O15C^ii^	96.62 (10)	O25W^iii^—K1—O7T	153.61 (9)
O13C—Cr2—O15C^ii^	83.38 (10)	O25W^iv^—K1—O7T	123.85 (9)
O15C—Cr2—O15C^ii^	180.00 (14)	O9T—K1—O7T	72.86 (8)
O13C^ii^—Cr2—O14C^ii^	83.45 (11)	O25W^iii^—K1—O26W^iv^	140.01 (9)
O13C—Cr2—O14C^ii^	96.55 (10)	O25W^iv^—K1—O26W^iv^	68.70 (9)
O15C—Cr2—O14C^ii^	96.19 (10)	O9T—K1—O26W^iv^	91.09 (9)
O15C^ii^—Cr2—O14C^ii^	83.81 (10)	O7T—K1—O26W^iv^	66.28 (8)
O13C^ii^—Cr2—O14C	96.55 (10)	O25W^iii^—K1—O23T	74.25 (8)
O13C—Cr2—O14C	83.45 (11)	O25W^iv^—K1—O23T	64.14 (8)
O15C—Cr2—O14C	83.81 (10)	O9T—K1—O23T	156.00 (8)
O15C^ii^—Cr2—O14C	96.19 (10)	O7T—K1—O23T	98.65 (8)
O14C^ii^—Cr2—O14C	180.000 (1)	O26W^iv^—K1—O23T	106.24 (9)
O8T—Mo1—O7T	106.84 (14)	O25W^iii^—K1—O24T^v^	91.73 (9)
O8T—Mo1—O4B	99.43 (12)	O25W^iv^—K1—O24T^v^	68.54 (8)
O7T—Mo1—O4B	100.45 (12)	O9T—K1—O24T^v^	70.82 (8)
O8T—Mo1—O6B^i^	101.16 (12)	O7T—K1—O24T^v^	110.61 (8)
O7T—Mo1—O6B^i^	96.54 (12)	O26W^iv^—K1—O24T^v^	57.72 (8)
O4B—Mo1—O6B^i^	148.14 (11)	O23T—K1—O24T^v^	132.52 (8)
O8T—Mo1—O1C	91.58 (12)	O25W^iii^—K1—O27W	83.91 (9)
O7T—Mo1—O1C	160.03 (12)	O25W^iv^—K1—O27W	134.40 (9)
O4B—Mo1—O1C	83.63 (10)	O9T—K1—O27W	84.97 (8)
O6B^i^—Mo1—O1C	71.79 (10)	O7T—K1—O27W	69.83 (8)
O8T—Mo1—O2C	159.33 (12)	O26W^iv^—K1—O27W	135.05 (9)
O7T—Mo1—O2C	93.25 (12)	O23T—K1—O27W	71.05 (8)
O4B—Mo1—O2C	71.53 (10)	O24T^v^—K1—O27W	153.77 (8)
O6B^i^—Mo1—O2C	80.83 (10)	O29W—K2—O17B	146.30 (10)
O1C—Mo1—O2C	69.32 (9)	O29W—K2—O19T^vi^	137.95 (10)
O10T—Mo2—O9T	105.54 (13)	O17B—K2—O19T^vi^	70.05 (8)
O10T—Mo2—O4B	98.88 (12)	O29W—K2—O8T^vii^	67.52 (10)
O9T—Mo2—O4B	104.05 (12)	O17B—K2—O8T^vii^	102.90 (8)
O10T—Mo2—O5B	98.79 (12)	O19T^vi^—K2—O8T^vii^	140.83 (9)
O9T—Mo2—O5B	95.15 (11)	O29W—K2—O28W	73.32 (10)
O4B—Mo2—O5B	149.26 (11)	O17B—K2—O28W	74.70 (8)
O10T—Mo2—O3C	93.09 (11)	O19T^vi^—K2—O28W	121.69 (9)
O9T—Mo2—O3C	157.67 (11)	O8T^vii^—K2—O28W	91.06 (9)
O4B—Mo2—O3C	84.69 (10)	O29W—K2—O10T^viii^	63.77 (10)
O5B—Mo2—O3C	69.42 (9)	O17B—K2—O10T^viii^	147.47 (8)
O10T—Mo2—O2C	161.78 (11)	O19T^vi^—K2—O10T^viii^	91.15 (8)
O9T—Mo2—O2C	92.28 (11)	O8T^vii^—K2—O10T^viii^	74.69 (8)
O4B—Mo2—O2C	72.55 (10)	O28W—K2—O10T^viii^	137.05 (8)
O5B—Mo2—O2C	83.06 (10)	O29W—K2—O30W^ix^	79.00 (15)
O3C—Mo2—O2C	70.46 (9)	O17B—K2—O30W^ix^	107.05 (14)
O11T—Mo3—O12T	106.13 (13)	O19T^vi^—K2—O30W^ix^	66.13 (12)
O11T—Mo3—O6B	100.61 (12)	O8T^vii^—K2—O30W^ix^	146.33 (14)
O12T—Mo3—O6B	102.43 (12)	O28W—K2—O30W^ix^	82.43 (11)
O11T—Mo3—O5B	100.44 (12)	O10T^viii^—K2—O30W^ix^	87.75 (13)
O12T—Mo3—O5B	92.82 (11)	O29W—K2—O9T^viii^	110.89 (10)
O6B—Mo3—O5B	149.33 (11)	O17B—K2—O9T^viii^	94.26 (7)
O11T—Mo3—O3C	92.56 (11)	O19T^vi^—K2—O9T^viii^	72.19 (8)
O12T—Mo3—O3C	156.92 (11)	O8T^vii^—K2—O9T^viii^	69.98 (8)
O6B—Mo3—O3C	86.90 (10)	O28W—K2—O9T^viii^	155.67 (8)
O5B—Mo3—O3C	70.11 (9)	O10T^viii^—K2—O9T^viii^	53.95 (7)
O11T—Mo3—O1C^i^	161.82 (11)	O30W^ix^—K2—O9T^viii^	121.81 (11)
O12T—Mo3—O1C^i^	91.92 (11)	Cr1—O1C—Mo1	103.93 (11)
O6B—Mo3—O1C^i^	72.66 (10)	Cr1—O1C—Mo3^i^	101.37 (11)
O5B—Mo3—O1C^i^	80.47 (10)	Mo1—O1C—Mo3^i^	91.53 (9)
O3C—Mo3—O1C^i^	70.51 (9)	Cr1—O2C—Mo2	102.54 (11)
O19T—Mo4—O20T	106.50 (14)	Cr1—O2C—Mo1	103.10 (10)
O19T—Mo4—O17B	104.09 (12)	Mo2—O2C—Mo1	92.00 (9)
O20T—Mo4—O17B	98.21 (12)	Cr1—O3C—Mo3	104.32 (11)
O19T—Mo4—O16B	94.09 (12)	Cr1—O3C—Mo2	103.08 (11)
O20T—Mo4—O16B	99.92 (12)	Mo3—O3C—Mo2	97.93 (10)
O17B—Mo4—O16B	149.41 (10)	Mo2—O4B—Mo1	119.12 (13)
O19T—Mo4—O15C^ii^	156.61 (11)	Mo2—O5B—Mo3	117.13 (12)
O20T—Mo4—O15C^ii^	93.29 (11)	Mo2—O5B—O16B^iii^	121.72 (12)
O17B—Mo4—O15C^ii^	84.78 (10)	Mo3—O5B—O16B^iii^	121.01 (12)
O16B—Mo4—O15C^ii^	69.81 (9)	Mo3—O6B—Mo1^i^	118.98 (13)
O19T—Mo4—O14C^ii^	91.56 (11)	Cr2—O13C—Mo5	102.58 (10)
O20T—Mo4—O14C^ii^	161.32 (12)	Cr2—O13C—Mo6	103.49 (11)
O17B—Mo4—O14C^ii^	72.15 (9)	Mo5—O13C—Mo6	92.48 (9)
O16B—Mo4—O14C^ii^	83.13 (9)	Cr2—O14C—Mo6	103.53 (11)
O15C^ii^—Mo4—O14C^ii^	70.30 (9)	Cr2—O14C—Mo4^ii^	102.33 (10)
O21T—Mo5—O22T	106.65 (13)	Mo6—O14C—Mo4^ii^	91.61 (8)
O21T—Mo5—O18B	99.32 (12)	Cr2—O15C—Mo5^ii^	103.82 (10)
O22T—Mo5—O18B	102.90 (12)	Cr2—O15C—Mo4^ii^	103.53 (10)
O21T—Mo5—O16B	100.17 (11)	Mo5^ii^—O15C—Mo4^ii^	97.60 (9)
O22T—Mo5—O16B	93.06 (11)	Mo4—O16B—Mo5	117.72 (11)
O18B—Mo5—O16B	150.02 (10)	Mo4—O17B—Mo6^ii^	118.91 (12)
O21T—Mo5—O15C^ii^	93.02 (11)	Mo5—O18B—Mo6	119.24 (12)
O22T—Mo5—O15C^ii^	156.22 (11)	H25A—O25W—H25B	96 (5)
O18B—Mo5—O15C^ii^	86.51 (10)	H26A—O26W—H26B	107 (5)
O16B—Mo5—O15C^ii^	69.93 (9)	H27A—O27W—H27B	108 (5)
O21T—Mo5—O13C	161.33 (11)	H28A—O28W—H28B	104 (5)
O22T—Mo5—O13C	91.69 (11)	H29A—O29W—H29B	121 (6)
O18B—Mo5—O13C	72.50 (10)	H30A—O30W—H30B	83 (7)
O16B—Mo5—O13C	81.93 (10)	H31A—O31W—H31B	107 (5)
O15C^ii^—Mo5—O13C	70.11 (9)	H32A—O32W—H32B	112 (5)

Symmetry codes: (i) −*x*+1, −*y*, −*z*+2; (ii) −*x*+1, −*y*+1, −*z*+1; (iii) −*x*+1, −*y*, −*z*+1; (iv) *x*−1, *y*, *z*; (v) −*x*, −*y*, −*z*+1; (vi) −*x*+2, −*y*+1, −*z*+1; (vii) −*x*+1, −*y*+1, −*z*+2; (viii) *x*+1, *y*+1, *z*; (ix) *x*+1, *y*, *z*.

### Hydrogen-bond geometry (Å, º)

**Table:**

*D*—H···*A*	*D*—H	H···*A*	*D*···*A*	*D*—H···*A*
O1*C*—H1···O22*T*^x^	0.70 (6)	2.11 (6)	2.789 (4)	164 (6)
O2*C*—H2···O27*W*	0.76 (6)	2.01 (6)	2.753 (4)	166 (6)
O3*C*—H3···O31*W*^xi^	0.82 (6)	1.79 (6)	2.604 (4)	176 (6)
O5*B*—H5···O16*B*^iii^	1.23 (1)	1.23 (1)	2.461 (3)	175 (5)
O13*C*—H13···O12*T*^iii^	0.70 (5)	2.05 (5)	2.734 (4)	167 (5)
O14*C*—H14···O32*W*	0.81 (5)	2.01 (5)	2.776 (4)	158 (5)
O15*C*—H15···O28*W*^ii^	0.76 (6)	1.87 (6)	2.619 (4)	170 (6)
O25*W*—H25*A*···O21*T*	0.92 (3)	1.96 (3)	2.838 (4)	157 (5)
O25*W*—H25*B*···O20*T*	0.93 (3)	1.92 (3)	2.819 (4)	162 (5)
O26*W*—H26*A*···O4*B*^i^	0.91 (3)	1.89 (3)	2.748 (4)	158 (5)
O26*W*—H26*B*···O24*T*^iii^	0.91 (3)	1.91 (3)	2.798 (4)	164 (5)
O27*W*—H27*A*···O18*B*	0.90 (3)	1.95 (4)	2.775 (4)	151 (5)
O27*W*—H27*B*···O32*W*	0.91 (3)	1.92 (3)	2.825 (5)	171 (5)
O28*W*—H28*A*···O12*T*^xii^	0.91 (3)	2.00 (4)	2.785 (4)	144 (5)
O28*W*—H28*B*···O31*W*^ii^	0.90 (3)	1.82 (3)	2.706 (4)	170 (5)
O29*W*—H29*A*···O11*T*^i^	0.95 (3)	2.11 (4)	2.949 (5)	146 (6)
O29*W*—H29*B*···O8*T*^vii^	0.96 (3)	2.18 (3)	3.126 (5)	169 (6)
O31*W*—H31*A*···O26*W*^ii^	0.90 (3)	1.77 (3)	2.665 (4)	170 (5)
O31*W*—H31*B*···O27*W*^ii^	0.92 (3)	2.18 (4)	2.952 (4)	141 (4)
O32*W*—H32*A*···O30*W*	0.89 (3)	2.06 (3)	2.947 (6)	171 (5)
O32*W*—H32*B*···O6*B*^i^	0.89 (3)	2.39 (5)	3.000 (4)	126 (5)

Symmetry codes: (i) −*x*+1, −*y*, −*z*+2; (ii) −*x*+1, −*y*+1, −*z*+1; (iii) −*x*+1, −*y*, −*z*+1; (vii) −*x*+1, −*y*+1, −*z*+2; (x) *x*, *y*, *z*+1; (xi) *x*, *y*−1, *z*+1; (xii) *x*, *y*+1, *z*.
